# Association with pathogenic bacteria affects life-history traits and population growth in *Caenorhabditis elegans*

**DOI:** 10.1002/ece3.1461

**Published:** 2015-03-23

**Authors:** S Anaid Diaz, Eric Q Mooring, Elisabeth G Rens, Olivier Restif

**Affiliations:** 1 Disease Dynamics Unit, Department of Veterinary Medicine, University of CambridgeMadingley Road, Cambridge, CB3 0ES, UK; 2 Delft Institute of Applied Mathematics, EEMCS Faculty, Delft University of TechnologyDelft, The Netherlands

**Keywords:** Development, diet, fitness, mathematical model, model system

## Abstract

Determining the relationship between individual life-history traits and population dynamics is an essential step to understand and predict natural selection. Model organisms that can be conveniently studied experimentally at both levels are invaluable to test the rich body of theoretical literature in this area. The nematode *Caenorhabditis elegans*, despite being a well-established workhorse in genetics, has only recently received attention from ecologists and evolutionary biologists, especially with respect to its association with pathogenic bacteria. In order to start filling the gap between the two areas, we conducted a series of experiments aiming at measuring life-history traits as well as population growth of *C. elegans* in response to three different bacterial strains: *Escherichia coli* OP50, *Salmonella enterica* Typhimurium, and *Pseudomonas aeruginosa* PAO1. Whereas previous studies had established that the latter two reduced the survival of nematodes feeding on them compared to *E. coli* OP50, we report for the first time an enhancement in reproductive success and population growth for worms feeding on *S*. *enterica* Typhimurium. Furthermore, we used an age-specific population dynamic model, parameterized using individual life-history assays, to successfully predict the growth of populations over three generations. This study paves the way for more detailed and quantitative experimental investigation of the ecology and evolution of *C. elegans* and the bacteria it interacts with, which could improve our understanding of the fate of opportunistic pathogens in the environment.

## Introduction

Free-living nematodes are recognized as major players in soil ecology (Freckman and Caswell 1985). Their ability to act as vectors and shed pathogenic bacteria in anthropogenic or arable soils has caused some concern for food production and public health (Gibbs et al. 2005; Locas et al. 2007). The bacterivorous nematode *Caenorhabditis elegans*, commonly found in anthropogenic organically enriched environments (Felix and Duveau 2012; Petersen et al. 2014), has been proposed as an experimental model to study the interactions of various bacteria with soil nematodes. Several studies have demonstrated that *C. elegans* can enhance the spread of pathogenic bacteria in the environment (Grewal 1991; Anderson et al. 2006). In addition, there is growing experimental evidence of fecal-oral transmission of bacteria within worm populations (Kenney et al. 2005; Diaz and Restif 2014). Importantly, the extent to which soil nematodes can contribute to bacterial persistence and spread will depend in large part on how these microbes affect the fitness of the worms they colonize.

Although the last 15 years have witnessed an exponential increase in the use of survival assays in *C. elegans* to assess the virulence of pathogenic bacteria (Marsh and May 2012), very few studies have actually measured the effects of pathogenic bacteria on the nematode's reproductive success (Coolon et al. 2009; MacNeil et al. 2013; Watson et al. 2013). It has been observed that while certain bacteria species increase nematode mortality during reproduction, others increase mortality post reproduction (Coolon et al. 2009), which could have very different effects on population growth. These observations suggest that time of reproduction and reproductive life span can be better predictors to assess the pathogenic effects of bacteria on population growth rather than survival only.

Despite its potential importance, there is no detailed information about the effects of opportunistic pathogens on nematode life-history traits and population dynamics. The virulence of bacteria in *C. elegans* has been mainly assessed by measuring worm mortality when feeding on any bacterial strain of interest, the reference being *Escherichia coli* OP50 (Darby 2005). In standard laboratory conditions, *C. elegans* feeding on *E. coli* OP50 has a median life span of around 12 days, compared to 6–11 days on *Salmonella enterica* Typhimurium SL1344 and 2–4 days on *Pseudomonas aeruginosa* PAO1 (Tan et al. 1999; Aballay et al. 2000; Portal-Celhay and Blaser 2012). As *C. elegans* reproduction takes place within 2–4 days of reaching adulthood, reduction in post-reproductive survival (as occurs for example on *S. enterica*) is not a useful predictor of fitness change. We recently reported that worms exposed for 24 h to *P. aeruginosa* PAO1 experienced a 40% reduction in fertility compared to worms fed on *S. enterica* Typhimurium (Diaz and Restif 2014), which raises questions about the relationship between survival and reproductive success. Although the pathogenicity or the low nutritional quality of strains might reduce both the life span and the fertility of nematodes, individuals can also respond to stress by shifting their investment from survival to reproduction (Alda Álvarez et al. 2005).

The aim of this study was to provide the first detailed quantification of the relative effects of *P. aeruginosa* PAO1, *S. enterica* Typhimurium, and *E. coli* OP50 on the life history and population growth of *C. elegans* in standard laboratory conditions. We proceeded in four steps, scaling up from individuals to populations. First, we measured the daily survival and reproduction of hermaphrodite nematodes feeding on each bacterial strain and kept either individually or in cohorts of 10 or 25; even though published studies often vary in the number of worms used in survival assays (ranging from 1 to 30 per plate), the effect of cohort size on life-history traits is hardly ever tested (Gems and Riddle 2000). Second, we recorded the timing of development, from eggs to adults, of offspring produced by individual hermaphrodite worms. The offspring were kept on the same bacterial strains as their respective parents. Third, we used these experimental data to parameterize an age-specific population dynamic model, which we used to predict the population growth of *C. elegans* on each bacterial strain in the absence of resource limitation. Finally, we tested these predictions experimentally by allowing individual worms to reproduce on bacterial lawns for 5 days, by which time three generations of nematodes would be coexisting. By integrating individual traits with population dynamics using an experimentally validated mathematical model, we hoped to demonstrate the potential of *C. elegans* as a model system for microbial ecology.

## Methods

### Strains and general maintenance

The N2 strain of *C. elegans* was obtained from the *Caenorhabditis* Genetic Centre (CGC) at the University of Minnesota and expanded using standard protocols (Hope 1999) for approximately four generations before storage at −80°C. Each experiment was started from a new cryopreserved stock and carried out at 25°C and on Petri dishes containing nematode growth media (NGM, US Biological, cat. No. N1000), and one of three bacterial strains: *E. coli* OP50-1 (streptomycin-resistant) provided by the CGC, *S. enterica* serovar *Typhimurium* JH3010 (chloramphenicol-resistant strain derived from wild-type strain SL1344) provided by Andrew Grant (University of Cambridge), and *P. aeruginosa* PAO1 (gentamycin-resistant strain) provided by Craig Winstanley (University of Liverpool). Before experiments, cryopreserved bacteria were thawed and grown for 16 h in Luria–Bertani (LB) broth at 37 °C; cultures were shaken at 220 rpm. LB media were supplemented with the appropriate antibiotic for selection (streptomycin 50 *μ*g/mL for *E. coli*, chloramphenicol 10 *μ*g/mL for *S. enterica,* and gentamycin 10 *μ*g/mL for *P. aeruginosa*). The average bacterial density per ml was *ca*. 1.99 × 10^9^ (SEM 9.89 × 10^6^), 1.97 × 10^9^ (SEM 6.46 × 10^6^), and 2.26 × 10^9^ (SEM 2.66 × 10^7^) for *E. coli*, *Salmonella,* and *P. aeruginosa*, respectively. Bacterial density was quantified by serial dilutions and quantification of viable bacteria (colony-forming units). NGM plates were seeded with 0.2 mL of liquid cultures.

### Nematode survival and reproduction

Reproduction and survival were determined using protocols adapted from Diaz et al. (2008). At the start of the experiment, we generated arrested first-stage larvae (L1) by hypochlorite treatment of eggs. After 24 h, larvae were transferred to fresh bacterial lawns either individually (25 replicates for each bacterial strain) or in cohorts of 10 (25 replicates) or 25 (10 or 11 replicates), marking the start of the experiment (time *t* = 0). Of the 1600 worms monitored, we detected five males, which were excluded from the analyses. Cohorts were transferred every day to fresh plates for the first 5 days (covering the whole reproductive period) and were monitored daily for survival until all worms had died. A worm was recorded as dead if it failed to respond to the touch of worm picker (Hope 1999). A small number of worms went missing during the experiment, which typically happens when they venture to the edge of the plate and die of desiccation, when they can no longer be seen. The overall proportion of worms that went missing was around 11%: 16 of 524 on *E. coli*, 50 of 548 on *P. aeruginosa,* and 58 of 527 on *S. enterica*. After each transfer, egg-containing plates (from which adults had been removed) were incubated for 48 h and the number of viable progeny on each day was counted. Hence, the lifetime reproductive success (LRS) was measured as the total number of viable eggs laid by an individual hermaphrodite or a cohort of worms. In addition, we calculated a potential LRS for each cohort, obtained by dividing the daily fecundity by the daily survival, in order to estimate the loss of fecundity caused by premature death of adult worms.

### Developmental time

Synchronized larvae were prepared as above and transferred individually to plates seeded with one of the three bacterial strains. After 48 h, adults were transferred to fresh plates (seeded with the same bacteria) for 2 h, before being removed. At this point, we counted the number of eggs laid during that 2-h window. All surviving larvae were monitored until they became adults. Individual plates were then monitored every 2 h during at least three time intervals: 0–14 h, 20–36 h, and 44–60 h. Worms reared in *P. aeruginosa* developed at a slower rate; therefore, plates were additionally monitored between 68 and 76 h. We recorded the abundance of four developmental stages: eggs, young larvae (L1–L3), fourth-stage larvae (L4), and adults.

### Population growth

As described above, synchronized larvae were transferred individually to NGM plates seeded with one of the three bacterial strains. These worms were allowed to grow and reproduce for 5 days without manipulation. The population size and composition on each plate (12 replicates per bacterial strain) were then assessed in two steps. First, the number of eggs, young larvae (L1, L2, or L3), older larvae (L4), and adults were determined directly by visual count through a dissecting microscope (a few plates were assessed three times to test the repeatability of the method). However, on plates seeded with *E. coli* or *S. enterica*, there were too many eggs and larvae for a correct assessment of these stages. Secondly, we used 1 mL of M9 buffer (Hope 1999) and a disposable bacteria spreader to wash and collect the contents of those plates into individual tubes. The volume of liquid recovered was measured to range from 0.6 to 0.75 mL. Each tube was vortexed, and two samples of 0.1 mL were taken and laid on glass slides into 10 drops to count the numbers of eggs, young larvae, L4, and adults present. A few tubes were counted thoroughly in order to calibrate the sampling procedure.

### Statistical analysis

#### Survival data

For the purposes of this study, we treated all causes of death (including “disappearance” as described above) equally, as they would all affect population dynamics equally. As mentioned before, the number of worms that went missing was low. Further, treating missing worms as censored data in the survival analysis did not affect the outcome (see Appendix S1, Table A1). As a result, the survival data were uncensored. We assessed the effects of food source and group size on mortality with Cox's proportional hazard model, using the Coxme package (Therneau et al. 2003) in R 3.0 (R Core Team 2013), and including a random plate effect for cohorts. The effects of food source and cohort size were assessed using the likelihood ratio test (LRT).

#### Lifetime reproductive success

We assessed the effect of food source on the LRS within each cohort size separately, using the Kruskal–Wallis test and adjusting the significance threshold value accordingly using Bonferroni method (significance threshold = 0.017). Next, we compared the LRS of cohorts of 10 or 25 worms with that of individual worms by bootstrapping: within each bacterial treatment, we randomly sampled the recorded LRS from 10 (resp. 25) individual worms with replacement, calculated the total number of offspring, and subtracted the number of offspring from one randomly selected cohort of 10 (resp. 25). This was repeated 100,000 times, and we computed the proportion of negative differences to estimate the probability that worms in cohorts of 10 (resp. 25) had lower LRS than individually reared worms.

#### Time of reproduction

We fitted a generalized linear model (GLM) to the LRS data in response to food source and cohort size. We pooled the daily records into early (viable eggs produced in the first 3 days) and late (day 4 or later) reproduction to simplify statistical treatment. The GLM was fitted using quasi-binomial error distribution (with a logit link function) to account for the large ratio between residual deviance and degrees of freedom (overdispersion).

#### Developmental time

Generalized mixed-effect models (GLMM) were used to analyze the changes in the proportion of developmental stages (egg, L1–3, L4, or Adult) across time and among food sources. We used a binomial error distribution (with a logit link function) for the model, including a random term describing the multiple worm developmental stages within plate and across time. The effects were assessed using the AIC model selection.

### Population dynamic model

We described the age-specific population dynamics with the McKendrick–von Foerster partial differential equation model (Cushing 1980):

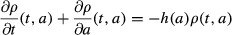


with boundary condition (birth term): 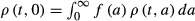, where *ρ*(*t*, *a*) is the age-dependent density function such that 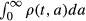 represents the total population size at time *t*, *h*(*a*) is the death rate at age a, and *f*(*a*) is the fecundity at age *a*. Age *a *=* *0 is that of an egg being laid. The death rate was calibrated as the hazard function of a Gompertz survival model (Lenaerts et al. 2007) fitted to each experimental survival assay (nine combinations of bacterial strains and cohort sizes), and the age-specific fecundity was calibrated by fitting Gamma distributions to the daily potential LRS from each experimental reproduction assay. We used data from the development assays to determine the effective age of synchronized larvae in the model, which provided the initial condition *ρ*(0, *a*). To compare the model predictions with the population growth experiments, we solved the partial differential equation numerically using the R package deSolve (Soetaert et al. 2010). We then integrated the continuous age distribution *ρ*(*t*, *a*) at *t *=* *5 days to obtain the numbers of eggs, young larvae, L4 larvae, and adults using the average molting ages measured experimentally with each bacterial strain in the developmental assays above. See Appendix S2 for complete description of the model implementation and analysis.

## Results

### Survival

**Figure 1:**
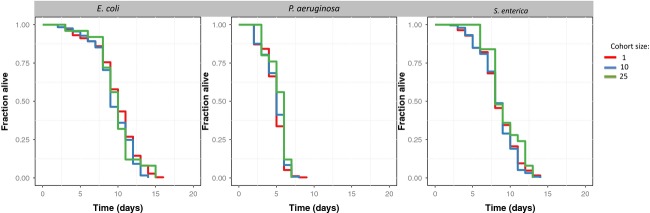
Survival of individuals in response to food source and cohort size. Lines represent the proportion of worms alive each day, aggregated across plates for cohorts of 1 (red lines), 10 (blue lines), or 25 (green lines). Each panel shows data from a different source of food: *E. coli* OP50 (left), *P. aeruginosa* PAO1 (center), and *S. enterica* JH3010 (right).

The median life span of individual worms (measured from L1 arrested development) was 10 days with *E. coli* OP50, 8 days with *S. enterica* JH3010, and only 6 days with *P. aeruginosa* PAO1 (Fig.1). According to Cox's proportional hazards model, food source, but not cohort size, had a significant effect on survival (Appendix S1, Table A1 *i–ii*). Similar results were obtained when including missing worms as censored individuals in the analysis (Appendix S1, Table A1 *iii–iv*). The analysis indicates that worms feeding on *E. coli* OP50 live nearly twice as long when feeding on *P. aeruginosa* PAO1 (odds ratio = 19.45 [95% CI: 15.74, 24.03] *P*-value < 0.001). The difference between worms feeding on *E. coli* OP50 and *S. enterica* JH3010 was smaller but yet significantly different (odds ratio = 1.70 [95% CI: 1.43, 2.02], *P*-value < 0.001).

### Lifetime reproductive success

**Figure 2:**
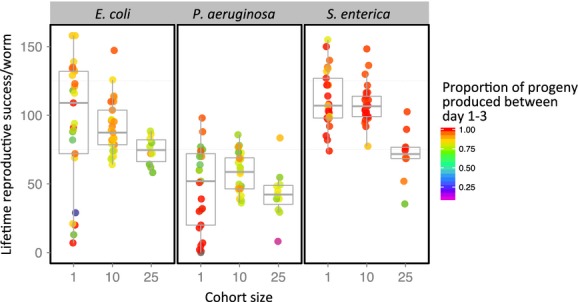
Lifetime reproductive success of *C. elegans* in response to food source and cohort size. Each dot represents a single cohort of worms: The position along the vertical axis shows the average reproductive success per worm, and the color indicates the proportion of the progeny laid by day 3 of the experiment (see Methods for details).

The median LRS of individual worms was 95, 48, and 112 on *E. coli* OP50, *P. aeruginosa* PAO1, and *S. enterica* JH3010, respectively (Fig.2). The effect of food source was statistically significant within each cohort size (*χ*^2^ = 32.73, 48.39, and 12.85, for 1, 10, and 25 cohort size, respectively, each with df = 2 and *P* < 0.001). Bootstrap analysis indicated that worms in cohorts of 25 had significantly lower LRS than individual worms when fed on *E. coli* OP50 (−25%) or *S. enterica* JH3010 (−36%), but not when fed on *P. aeruginosa* PAO1; in contrast, cohorts of 10 exhibited no significant loss or gain in reproduction compared to individuals (Table1). We then assessed the effect of mortality on reproductive success by taking into account the potential contribution of those worms reported dead (LRS_Potential_, Table1). The results suggest that mortality had little effect on the fecundity of worms feeding on *E. coli* OP50 and *S. enterica* JH3010, as most of them died long after they finished reproducing. However, early mortality among worms feeding on *P. aeruginosa* PAO1 reduced fecundity between 8 and 18% depending on the cohort size (Table1).

**Table 1:** Variation in LRS in relation to food source and cohort size. Table shows the mean and standard deviation of the effective LRS, which is the sum of viable offspring, and the potential LRS, which takes into account the potential contribution of those worms that died during the reproductive period, and the relative loss (1 − LRS_Effective_/LRS_Potential_)

Food source	Cohort size	*N*	LRS_Effective_	LRS_Potential_	Loss
			Mean ± SD	Mean ± SD	Mean
*Escherichia coli* OP50	1	25	95.20 ± 46.09	95.76 ± 45.21	0.02
	10	25	91.54 ± 20.04	93.43 ± 19.34	0.02
	25	10	74.35 ± 10.41	75.93 ± 10.61	0.02
*Pseudomonas aeruginosa* PAO1	1	24	48.75 ± 29.19	49.44 ± 30.69	0.08
	10	25	58.31 ± 14.58	69.50 ± 11.36	0.16
	25	11	42.72 ± 18.50	52.43 ± 20.04	0.18
*Salmonella enterica* JH3010	1	25	112.04 ± 21.15	112.04 ± 21.16	0
	10	25	108.34 ± 14.80	109.67 ± 14.64	0.01
	25	10	71.30 ± 18.44	72.83 ± 19.46	0.02

### Timing of reproduction

**Figure 3:**
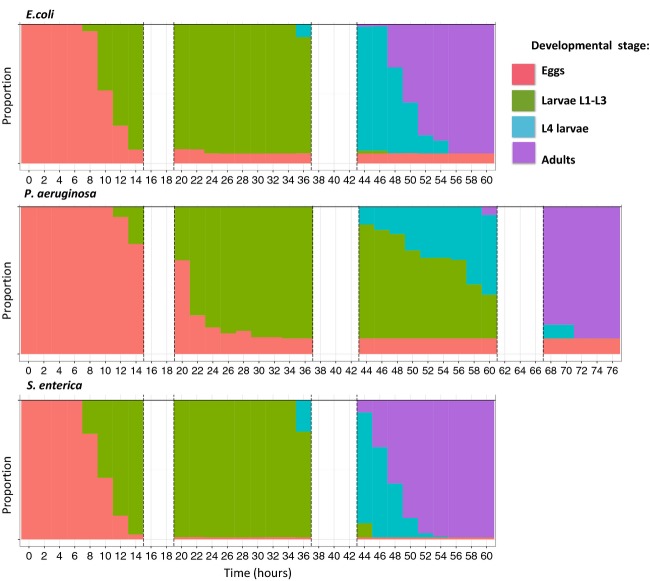
Timing of nematode development, broken down by food source, and starting from eggs laid within a 2-h window by individually reared hermaphrodites. In each row, stacked bars represent the proportions of eggs, larvae L1–L3, larvae L4, and adults across time (red, green, blue, and purple, respectively). Blank areas were times when worms were not monitored.

Across all groups, reproduction ceased within 6 days, with a peak recorded on the third day on most plates (Appendix S1, Fig. A1). For simplicity, we classified eggs laid within 3 days as “early reproduction” and the remaining as “late reproduction”. Although there was a high variability within experimental groups, the GLM analysis indicated a significant effect of food source and cohort size on the way worms spread their offspring, with no evidence of interaction between the two factors (Appendix S1, Table A2). On average, individuals fed on *P. aeruginosa* PAO1 laid their eggs later, with 20% of “late reproduction”, compared to 16% on *E. coli* OP50 and only 5% on *S. enterica* JH3010 (Fig.3).

### Development

According to the GLMM analysis, food source significantly affected the timing of development (Appendix S1, Table A3). Eggs from mothers fed on *P. aeruginosa* PAO1 had the slowest development (median hatching time 29 h, almost three times as long as on the other strains) as well as the highest hatching failure rate (14% after 4 days) (Fig.3). The median time from an egg being laid to adult stage was 50 h on *E. coli* OP50, 65 h in *P. aeruginosa* PAO1, and 48 h in *S. enterica* JH3010.

### Population growth

**Figure 4:**
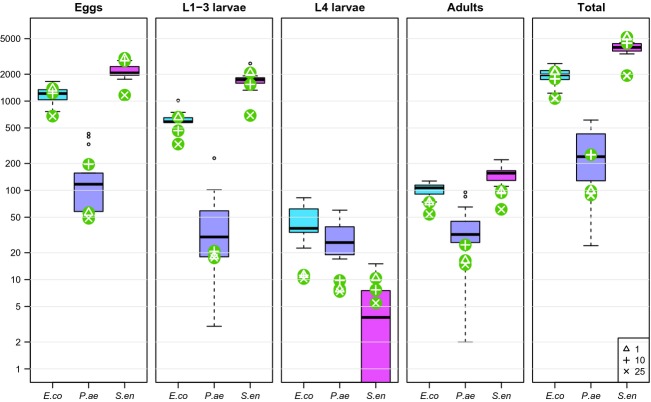
Comparison of predicted and observed population growth after 5 days. Boxplots show distributions of stages on plates for each bacterial strain (*E.co = E. coli* OP50, *P.ae = P. aeruginosa* PAO1, *S.en = S. enterica* JH3010). Overlaid symbols indicate values predicted by the population dynamic model parameterized with life-history data from each experimental group (cohort sizes: **Δ** = 1, **+** = 10, **× **= 25).

When allowing individual larvae to grow and reproduce untouched for 5 days, the population sizes (including eggs) reached on average 1970 on *E. coli*, 274 on *P. aeruginosa,* and 4080 on *S. enterica*. The numbers of eggs, young larvae (L1 to L3), and adults followed the same order, whereas the number of L4 larvae was lowest on *S. enterica* (Fig.4). These patterns were consistent with the numbers predicted from the population dynamic model fitted to life-history assays (Fig.4). In particular, parameter estimates obtained from cohorts of 10 worms led to predicted total population sizes within 10% of the observed ones: 1772 with *E. coli*, 250 with *P. aeruginosa,* and 4456 with *S. enterica*. However, the fitted models tended to underestimate the L4 and adult stages (Fig.4), potentially reflecting the shorter window of development of L4s stage compared to the other developmental stages (L1–L3 and adults, Fig.3). We found that small changes in developmental time can have a large impact in the predicted number of L4 worms (Appendix S2, Fig. B5).

## Discussion

### Bacteria and reproductive success

This study provides the first detailed account of the quantitative effects of different species of bacterial pathogens on the life history and population growth of the free-living nematode *C. elegans*. In addition to previously reported reductions in life span, we found that the two opportunistic pathogens *Salmonella enterica* Typhimurium and *Pseudomonas aeruginosa* (PAO1) induced substantial variations in the development, timing of reproduction and lifetime reproductive success of *C. elegans* N2 compared to the standard source food *E. coli* OP50. Furthermore, we showed, both numerically and experimentally, that these combined variations in life-history traits led to contrasted population dynamics: within 5 days, single worms feeding on *S. enterica* produced 15 times as many offspring (across two generations) as those feeding on *P. aeruginosa*, and twice as many as those feeding on *E. coli* OP50. The latter finding was unexpected: most studies of the pathogenesis of *S. enterica* in *C. elegans* have only reported reductions in survival compared to worms fed on *E. coli* OP50 (e.g., Aballay et al. 2000; Tenor et al. 2004; Portal-Celhay and Blaser 2012), concluding that *S. enterica* is in effect a pathogen for *C. elegans*. In our experiment, however, the shorter life span was more than made up for by faster development and reproduction and higher reproductive success.

Worms fed on *P. aeruginosa* PAO1 experienced lower survival and fecundity than the other groups, coupled with a slower developmental time. We estimated that nematodes fed on PAO1 lost up to 20% of their reproductive success due to early mortality. However, this only accounted for a small portion of the reduction in fecundity compared to that of worms fed on *E. coli* OP50. *P. aeruginosa* PAO1′s combination of multiple negative effects resulted in a considerably slower population growth compared to both *S. enterica* JH3010 and *E. coli* OP50.

Our results also demonstrate that variations in cohort size from 1 to 25 do not influence survival assays. In contrast, cohorts of 25 worms experienced much lower reproductive success than worms kept individually or in cohorts of 10. We have reasons to believe that food limitation did not play any substantial role in this discrepancy. First, worms were transferred to fresh plates every day, and hence, their eggs were spread on several plates, reducing the competition among larvae: There were never more than 1000 worms per plate. Second, we did not observe any sign of starvation following incubation of egg-containing plates for 48 h; specifically, the food was not depleted and worms showed no signs of internal hatching (Chen and Caswell-Chen 2004). Third, the population growth experiment demonstrated that worms can grow on a single plate for 5 days, accumulating two generations of offspring, without showing any sign of resource limitation. As we did not directly record the numbers of eggs laid daily in the reproduction assay, we can only hypothesize that hermaphrodites kept in cohorts of 25 must have laid either fewer eggs overall or fewer viable eggs. The latter could explain why we did not observe any apparent reduction in reproductive success at the end of the population growth assay: By day 5, most of the “grandchildren” of the initial worms were still in eggs, as predicted by the model (Fig.4), but we did not assess their viability. So we cannot exclude the possibility that the first generation of offspring, as they matured in cohorts of several dozens, experienced density-dependent effects similar to those we had observed in cohorts of 25, and laid eggs of lower quality. In the absence of food limitation, our leading hypothesis is that social interactions within cohorts, for example pheromone manipulation, may have contributed to the observed reduction in lifetime reproductive success (Gems and Riddle 2000; Artyukhin et al. 2013; Ludewig et al. 2013). Further experimental work will be needed to investigate this phenomenon.

### From life-history traits to population growth

To our knowledge, this is the first attempt to parameterize and validate an age-specific population dynamic model for *C. elegans*. Although the genetic and environmental drivers of aging, reproduction and development in *C. elegans* have been extensively analyzed for many years (Klass 1977), few studies have considered the implications for population dynamics and ecology (Hodgkin and Barnes 1991). Life-history tables, which compile survival and reproductive success at regular intervals, are one of the most common tools used by ecologists to predict population growth (Birch 1948) as well as life-history evolution (Brommer 2000). These methods have been applied to several *Caenorhabditis* species grown in the laboratory (Diaz et al. 2008; Muschiol et al. 2009; Lancaster et al. 2012; Darby and Herman 2014), but the resulting estimates of population growth rates have never been tested experimentally. The growing interest in the natural ecology of *C. elegans* (Félix and Braendle 2010) and the reappraisal of this organism as a powerful model system for experimental evolution (Gray and Cutter 2014) highlight the need for more detailed characterization of the population dynamics of *C. elegans*. Our results highlight the limitations of survival alone to predict population dynamics and therefore the evolution of *C. elegans* life history. We observed that an earlier mortality of worms feeding on *S. enterica* had little effect on population growth, compared to an *E. coli* diet; in contrast, shifts in development and reproduction can affect population growth. Our work provides the experimental framework and a fitness baseline for evaluating evolutionary changes. This study demonstrates how simple experimental procedures can be combined with mathematical models to bridge the gap between individual traits, population dynamics, and evolution.

### Bacterial duality

The mechanisms that contribute to the variation in worm survival, reproduction, and development in response to different strains of live bacteria are not fully understood and likely to be complex. The complexity is related to the fact that these bacteria act simultaneously as food and symbionts of *C. elegans* (reviewed by Kim 2013). Possible ways to start disentangling the two effects would be adding drugs that inhibit bacterial proliferation and/or feeding killed bacteria to nematodes (Evason et al. 2005). Although some bacterial strains have been described as pathogenic to *C. elegans* on the basis that worms fed on killed bacteria survive longer than those fed on live bacteria (Aballay et al. 2000; Couillault and Ewbank 2002), this does not always correlate with changes in reproduction or development (MacNeil et al. 2013). Several bacterial pathways and metabolites have been shown to affect the survival of *C. elegans* on *S. enterica* (Tenor et al. 2004), *P. aeruginosa* (Zaborin et al. 2009), and *E. coli* (Virk et al. 2012), which themselves can depend on environmental factors (Zaborin et al. 2009; Cezairliyan et al. 2013). The results presented here indicate that early mortality caused by *P. aeruginosa* PAO1 was only partly responsible for the reduction in worm fecundity; we are now testing multiple mutants of *P. aeruginosa* to see whether virulence factors previously linked to mortality in *C. elegans* also affect other life-history traits.

A factor overlooked by most studies looking for bacterial virulence factors in *C. elegans* is the effect of bacterial abundance and growth inside its host; indeed, these variables are hardly ever reported. We recently showed that adult worms infected with *P. aeruginosa* PAO1 harbor 10 times as many bacteria as worms of the same age infected with *S. enterica* JH3010 (Diaz and Restif 2014). Portal-Celhay and Blaser (2012) measured in vivo competition between *E. coli* OP50 and several *S. enterica* strains. Although it has been shown that iron acquisition by *P. aeruginosa* accelerates nematode death (Kirienko et al. 2013), more research is needed to determine the importance of competition for essential resources between *C. elegans* and the bacteria that colonize its intestine, and what abiotic changes directly or indirectly affect worms and bacteria. In our study, there were not environmental factors inhibiting bacterial proliferation on the plate. However, we used three different selective antibiotics for growing bacterial broths, small quantities of which ended up on the agar plates along with the bacterial broth. To our knowledge, streptomycin, chloramphenicol, and gentamycin have not been reported to directly affect worm fitness (see review Lucanic et al. 2013). Additionally, the concentration of each antibiotic was lower than drug concentrations that are known to affect worm development (ours: <45 *μ*mol/L and others: 3–50 mmol/L – e.g., Onken and Driscoll 2010; Evason et al. 2005). Further research is needed to uncouple the nutritional and pathogenic effects of bacteria in abiotic conditions.

### Relevance for microbial ecology

Recent observations have suggested that *C. elegans* can contribute to bacterial persistence by spreading bacteria in the environment and enhancing nematode infection (Kenney et al. 2005; Diaz and Restif 2014). We previously demonstrated that *P. aeruginosa* achieves higher transmission success than *S. enterica* within experimental populations of worms (Diaz and Restif 2014). However, the negative effects of *P. aeruginosa* on worm population dynamics are likely to limit the spread of these bacteria by *C. elegans*. In contrast, the higher fitness of nematodes on *S. enterica* could have a positive feedback on the bacteria population dynamics.

An important caveat is that these assays have been carried out on an artificial growth medium, which may not reflect natural environments. Although *Caenorhabditis* worms thrive in rich compost (Felix and Duveau 2012), much less is known about their interactions with bacteria in harsher environments. For example, worms grown on soil substrates have lower survival (Van Voorhies et al. 2005) and fecundity (Freyth et al. 2010) than on NGM plates. Upon food depletion, young larvae of *C. elegans* can enter a long-lived and arrested reproductive development called dauer larvae stage (Félix and Braendle 2010). This developmental stage is commonly found in association with invertebrates and it is considered the dispersal stage (Félix and Braendle, Felix and Duveau 2012). To what extent dauer larvae development can contribute to environmental bacterial growth and consequently enhance nematode expansion remains to be answered. For the insect-parasitic nematode *Rhabditis blumi*, dauer larvae carrying bacteria can cause higher mortality in wax moth larvae compared to axenic juveniles (Park et al. 2011).

At a time when host–symbiont interactions are increasingly studied at the molecular level, it is essential to develop complementary tools to quantify the growth of the organisms themselves. Together with our recent measurements of bacterial colonization and transmission in cohorts of *C. elegans*, this study paves the way for more detailed investigations of the ecology of free-living nematodes and the bacteria they both feed on and harbor. The association between opportunistic bacterial pathogens and nematodes can have important implications for food security and public health, making *C. elegans* a useful experimental model for microbial ecology.

## Supporting Information

**Appendix S1.** Supplementary Results for the analysis of life-history traits.

**Appendix S2.** Population dynamic model.
