# Supplementary material for: Association with pathogenic bacteria affects life-history traits and population growth in Caenorhabditis elegans
Source: Ecol Evol. 2015 Mar 23;5(8):1653–63. doi: 10.1002/ece3.1461 (PMC4409413; doi:10.1002/ece3.1461)
Supplement: Supplementary file 1 [file ece30005-1653-sd1.pdf]

Appendix A. Supplementary Results for the analysis of life-history traits.

S.A. Diaz, E.Q. Mooring, E.G. Rens and O. Restif

Table A1. Cox's proportional hazard survival models. Model comparison of survival models when *i*) including all worms (uncensored data) and *iii*) when considering missing worms as censored. Model comparison was conducted using the likelihood ratio test. Columns indicate log likelihood estimate (loglik), χ^2-value, Degrees of freedom (Df) and P-value (best model shown in bold). *ii* and *iv* describe the statistics of the preferred models. The fixed effect terms are the hazard coefficients of *P. aeruginosa* and *S. enterica* in comparison to *E. coli*. The random effects indicate the variation among individuals within plates.

i) Uncensored data	loglik	χ^2	Df	P-value
Null model	-9788.2			
**Food source**	**-9631.1**	**314.3**	**2**	**< 0.001**
Food source + cohort size	-9629.2	3.84	2	0.15
Food source * cohort size	-9627.5	3.38	4	0.49
ii) Best model statistics:				
Fixed effects	Coefficient	Std. error	z-value	p-value
P. aeruginosa	2.97	0.11	27.51	< 0.001
S. enterica	0.53	0.09	6.00	< 0.001
Random effects			Std.Dev.	Variance
Individual within plate			0.26	0.07
iii) Censored data	loglik	χ^2	Df	P-value
Null model	-9788.2			
**Food source**	**-9631.1**	**314.3**	**2**	**< 0.001**
Food source + cohort size	-9631.1	0.02	2	0.88
Food source * cohort size	-9629.6	2.89	4	0.32
iv) Best model statistics:				
Fixed effects	Coefficient	Std. error	z-value	p-value
P. aeruginosa	2.96	0.10	27.51	< 0.001
S. enterica	0.53	0.08	6.00	< 0.001
Random effects			Std.Dev.	Variance
Individual within plate			0.26	0.07

Table A2. Analysis of deviance for generalised linear models describe the proportion of early reproduction, using a quasibinomial error distribution (see Methods). Best model shown in bold.

Models	Residual deviance	Df	Deviance	F-value	P-value
Null model	11067.9				
Food source	5372.8	2	5695.2	94.6394	< 0.001
**Food source + Cohort size**	**4628.1**	**2**	**744.7**	**12.3745**	**< 0.001**
Food source * Cohort size	4363.3	4	264.8	2.1998	0.071

Table A3. Comparison of generalized mixed-effect models to describe the effect of food on the development of nematodes, taking into account repeated measurements within cohorts. Best model shown in bold according to Akaike's Information Criterion (AIC). It also describes the log likelihood value (logLik), χ^2 –value, degrees of freedom (Df) and P-vale

Models	AIC	logLik	χ^2	Df	P-value
Eggs					
Null model	3773.0	-1884.5			
Food source	2030.5	-1012.3	1744.4	1	< 0.001
Food source + Cohort size	2013.2	-1001.6	21.3	2	< 0.001
**Food source * Cohort size**	**1782.4**	**-884.2**	**234.7**	**2**	**<0.001**
Larvae					
Null model	4388.8	-2129.4			
Food source	4216.4	-2105.2	174.4	1	< 0.001
Food source + Cohort size	4217.4	-2103.7	3.02	2	0.22
**Food source * Cohort size**	**4197.9**	**-2075.8**	**4151.6**	**2**	**<0.001**
L4					
Null model	2560.4	-1278.2			
Food source	2146.2	-1070.1	416.2	1	< 0.001
Food source + Cohort size	2133.9	-1062.0	16.3	2	<0.01
**Food source * Cohort size**	**2129.3**	**-1057.7**	**8.65**	**2**	**<0.05**
Adults					
Null model	4001.6	-1998.8			
Food source	1141.0	-567.5	2862.5	1	< 0.001
Food source + Cohort size	1042.4	-516.19	102.67	2	<0.01
**Food source * Cohort size**	**1037.2**	**-511.6**	**9.20**	**2**	**<0.05**

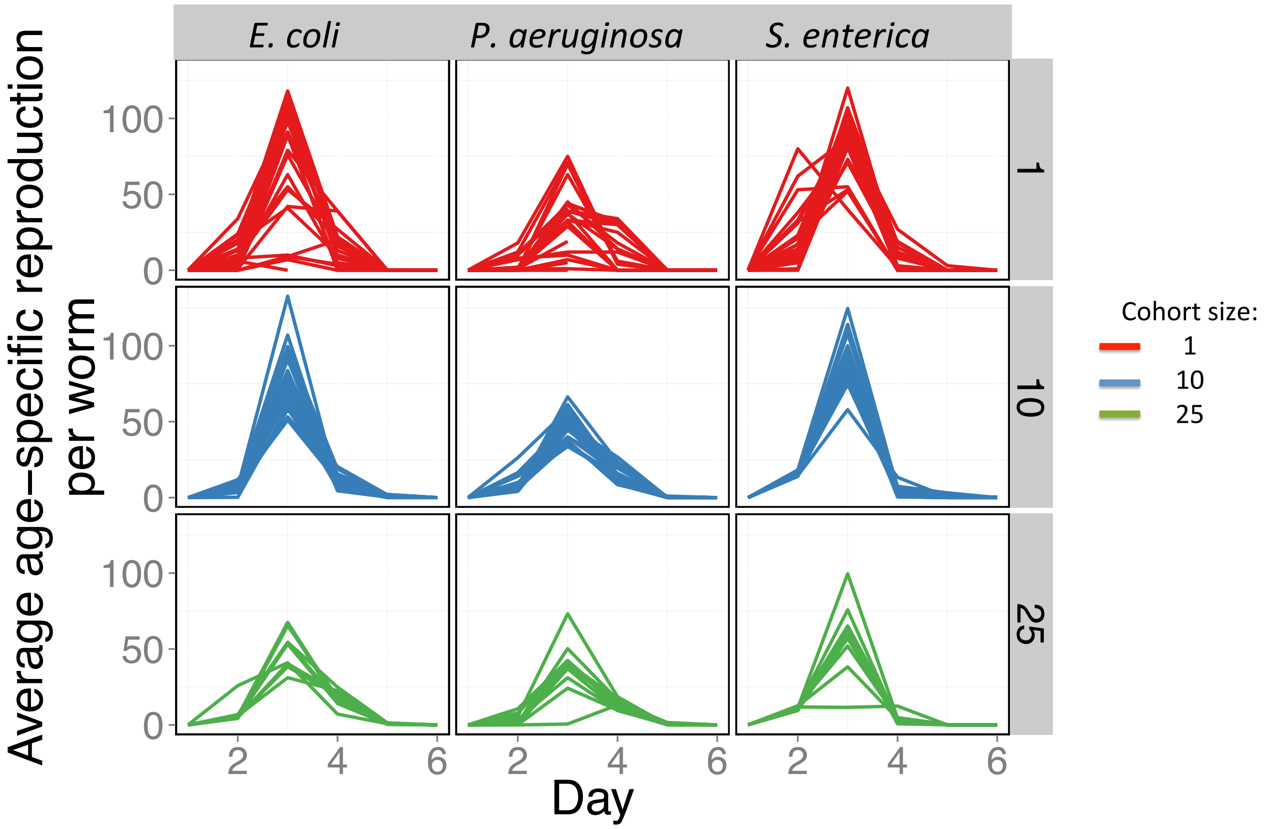

Figure A1. Time-series of the average number of viable eggs per worm laid each day. Each panel shows an experimental group, and each line follows one cohort.
