# Supplementary material for: Association with pathogenic bacteria affects life-history traits and population growth in Caenorhabditis elegans
Source: Ecol Evol. 2015 Mar 23;5(8):1653–63. doi: 10.1002/ece3.1461 (PMC4409413; doi:10.1002/ece3.1461)
Supplement: Supplementary file 2 [file ece30005-1653-sd2.pdf]

Appendix B. Population Dynamic Model

S.A. Diaz, E.Q. Mooring, E.G. Rens and O. Restif

This Appendix describes how the population dynamic model was parametrized and implemented.

Abbreviations in tables and figures: Eco = *E. coli*, Pae = *P. aeruginosa*, Sen = *S. enterica*.

1. Supplementary Methods

In the following sections, we use t to denote the time of the relevant experiment, and a the age of a worm from the egg-laying time. In the survival and reproduction assays and in the population growth assay, experiments were started ($t = 0$) when arrested L1 larvae were transferred to bacterial lawns. First, we parametrize a hazard function (death rate) and a reproduction function with respect to the experimental time t. Second, we determine the effective initial age $a(t = 0)$ by comparing the time to adult stage for synchronised larvae and for eggs measured in developmental assays. Third, we define the age-specific birth rate $\beta(a)$ and death rate $\mu(a)$ by adding the corresponding time shift.

1.1. Parametric Survival analysis

Definitions

- Lifetime duration: T
- Lifetime pdf: $f(t)$
- Lifetime cdf: $F(t) = P(T \leq t) = \int_0^t f(u)du$
- Survival function: $S(t) = P(T > t) = 1 - F(t)$
- Hazard function: $h(t) = \frac{f(t)}{S(t)} = \lim_{dt \to 0} P(T < t + dt \,|T \geq t)$
- Cumulative hazard function: $H(t) = \int_0^t h(u)du = -\log S(t)$

Let n_i be the number of worms observed to be alive at time t_i, starting at t_0. In the absence of censoring, the Kaplan-Meyer estimator of the survival function is equal to the proportion of worms still alive at a given time:

$$\hat{S}(t_k) = \prod_{0<i\leq k} \frac{n_i}{n_{i-1}} = \frac{n_k}{n_0}$$

Parametric hazard function For the development of the population dynamic model, where the age-specific death rate corresponds to the hazard function, we fit $h(t)$ to the survival data, assuming that it follows a standard mathematical function. In line with previous work (see main text), we chose a Gompertz model, which assumes that the hazard increases exponentially with age: $h(t) = \beta\,\gamma\,e^{\beta\,t}$, with scale $\beta > 0$ and shape γ, which leads to survival $S(t) = \exp\{-\gamma(e^{\beta t} - 1)\}$ and to the pdf $f(t) = \beta\,\gamma\,\exp\{\gamma + \beta\,t - \gamma\,e^{\beta\,t}\}$.

Maximum-likelihood fitting of survival functions Given a survival function $S(t)$ with a set of parameter $\Theta = \beta, \gamma$, the likelihood of recording a death at time t_i is $l_{t_i}(\Theta) = P(t_{i-1} < T \leq t_i) = S(t_{i-1}) - S(t_i)$

With daily observations, the likelihood of recorded times of death $\{t_1, ..., t_n\}$ is $L(\Theta) = \prod_{i=1}^n S(t_i - 1) - S(t_i)$

The goodness-of-fit was assessed in two ways: first by running a discrete Cramer-von Mies test using the **dgof** package (Arnold and Emerson 2011), second by Monte Carlo simulations of observed times of death drawn randomly from the fitted Gompertz distributions.

1.2. Developmental assays

In addition to the developmental assay (D_{egg}) presented in the main text (Fig. 3), which was started from eggs, we ran a similar assay (D_L) starting from synchronised larvae. Arrested L1 larvae were generated as described in the main Methods, and transferred individually to bacterial lawns (t=0). Their status was recorded every two hours from t=0 to t=26 h, and again from t=34 to t=48 h. For each source of food, we then estimated the effective age a_0 of synchronised larvae at t=0 as the difference between the median time to Adult stage t_A (as per D_L) and the median age a_A at which egg cohorts reached the adult stage (as per D_{egg}). For example, if the median time from synchronised L1 to Adult was $t_A = 30$ h and the median time age at Adult stage was $a_A = 36$ h, we would infer that synchronised larvae were effectively 6 hour-old at the start of the experiment.

1.3. Timing of egg-laying

In the life-history experiments, fecundity at time t was measured as the number of viable larvae from eggs laid between t-1 and t. The fecundity of cohorts was pooled across all adult worms on a plate that were alive at time t-1. As explained in the main text, the recorded number of viable offspring (LRS) was occasionally reduced by early death of mothers. However, the population dynamic model requires that we use the *potential* LRS that would be achieved in the absence of maternal death. This variable, which we denote as R, was computed for each experimental group by assuming that worms that died between day t and $t+1$ achieved on average half of their potential fecundity on that day.

We model egg-laying as a continuous stochastic process, assuming egg-laying events are i.i.d. with probability density function $g(t)$. The predicted proportion of the LRS laid on day t is $\int_{t-1}^{t} g(u)du = G(t) - G(t-1)$ where $G(t) = \int_0^t g(u)du$. For a given parametric model $g(t;\Theta)$, and daily reproductive success time-series $x_1, ..., x_k$ from a single worm, the likelihood is given by a multinomial distribution, hence the expression for the log-likelihood: $\log L_w(\Theta) = \sum_{i=1}^{k} x_i \log[G(i;\Theta) - G(i-1;\Theta)]$. The same applies to cohorts of 10 or 25 worms, considering a cohort as a single "super-worm" with a larger number of eggs. Assuming the worms and plates in a given experiment are i.i.d, we can just sum the individual log-likelihoods.

For convenience we assumed that age-specific egg-laying followed a Gamma distribution from the time worms become adults. We used the median value of t_A as defined in the previous section, and imposed $g(t) = 0 \forall t < t_A$. In order to determine fecundity at age a, we use the parametrized function $g(t)$ at time $t = a - a_0$, where a_0 is the effective age at $t = 0$ as per the previous section. More specifically, the age-specific birth rate $\beta(a)$ was obtained by multiplying $g(a - a_0)$ by the potential reproductive success R. We parametrized the model for each experimental group using the average of R (Table 1) to produce Fig. 4 in the main text. Below we also present results from simulations obtained by varying R in line with data from the life-history assays.

1.4. Population dynamic model

Let $\rho(t, a)$ be the age-density function of the population at time t: by definition $\int_{a_1}^{a_2} \rho(t, a)da$ represents the number of individuals in the population with ages in $[a_1, a_2]$ at time t. The total population size at time t is $N(t) = \int_0^\infty \rho(t, a)da$.

Let $\beta(a) = Rg(a - a_0)$ be the age-specific birth rate, with R and g as defined above. Then $\int_{a_1}^{a_2} \beta(a)\rho(t, a)da$ is the number of eggs laid by individuals with ages in $[a_1, a_2]$ per unit time. The total birth rate at time t is $B(t) = \int_0^\infty \beta(a)\rho(t, a)da$.

Let $\mu(a) = h(a - a_0)$ be the age-specific death rate, then $\int_0^\infty \mu(a)\rho(t, a)da$ is the total death rate.

The population dynamics follows the Lotka-McKendrick model:

$$\frac{\partial \rho}{\partial t}(t, a) = -\mu(a)\rho(t, a) - \frac{\partial \rho}{\partial a}(t, a)$$

with boundary conditions: $\rho(t,0) = B(t), \forall t > 0; \rho(0,a) = \phi(a), \forall a > 0$, where the initial age distribution $\phi(a)$ is given by the experimental design. All experiments were started at time $t = 0$ by transfering arrested L1 larvae on food. Thus, we can assume a narrow initial age distribution around a_{L1}.

The birth and death rate functions $\beta(a)$ and $\mu(a)$ were chosen in line with analysis of individual and cohort dynamics. Life-history experiments were initiated at $t = 0$ with synchronised L1 larvae, hence $a_{L1} < a(t = 0) < a_{L2}$. If $\hat{h}(t)$ is the fitted hazard function for survival, we must use death rate $\mu(a) = \hat{h}(a - a_0), \forall a > a_0$; the survival of eggs is unknown. The fecundity was measured by counting viable offspring, so it accounts any egg mortality. Therefore we can assume in the model that $\mu(a) = 0, \forall a < a_{L1}$. In addition, we impose $\beta(a) = 0, \forall a < a_{adult}$. The partial differential equation was then solved numerically using the ode.1D function from the **deSolve** package in R.

---

2. Supplementary Results

2.1. Gompertz survival model

Table B1. Maximum likelihood estimates for the shape and scale parameters of the Gompertz survival functions for each experimental group.

food	size	shape	scale
Eco	1	0.01840	0.386
Eco	10	0.00824	0.476
Eco	25	0.01311	0.410
Pae	1	0.00482	1.004
Pae	10	0.01748	0.809
Pae	25	0.02111	0.791
Sen	1	0.01195	0.458
Sen	10	0.01805	0.454
Sen	25	0.02488	0.411

Table B2. Results of a Cramer-von Mies test of goodness-of-fit for the Gompertz model: lower P-values indicate greater discrepancy between predicted and observed values.

food	size	P
Eco	1	0.2777
Eco	10	0.0517
Eco	25	0.2333
Pae	1	0.6244
Pae	10	0.1569
Pae	25	0.1446
Sen	1	0.2552
Sen	10	0.1655
Sen	25	0.1035

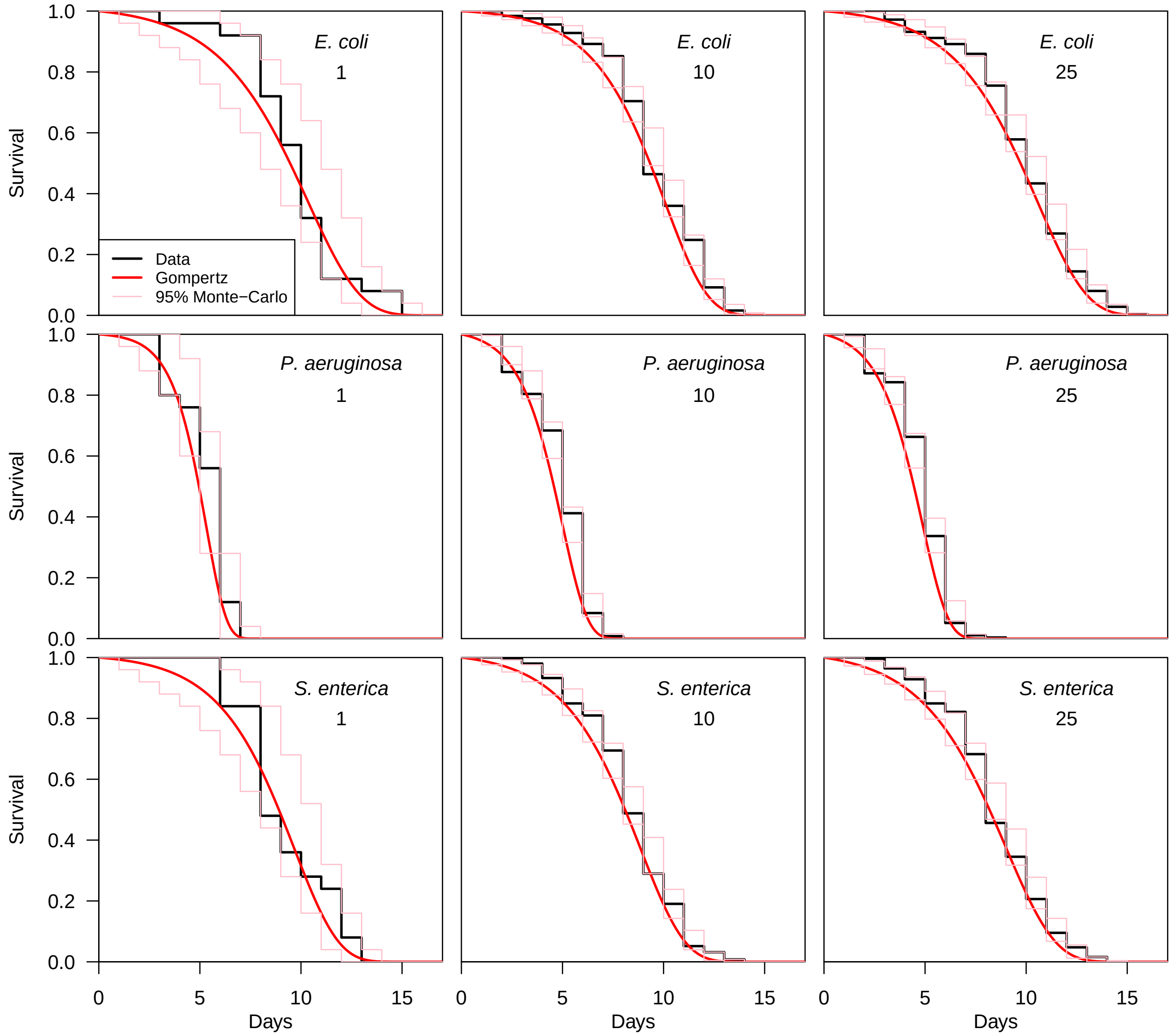

Figure B1. Each panel shows the daily proportion of worms alive in each experimental group (black lines), the fitted Gompertz model (red curves), and the 2.5 and 97.5 percentiles of simulated distributions of lifespans from the fitted Gompertz survival functions for each group, using the same number of observations as in the experimental data (pink lines).

2.2. Developmental assays

Table B3. Median age (in days since egg-laying) of synchronised larvae and key developmental transitions on each food source

	Sync	Hatching	L4	Adult
Eco	0.486	0.420	1.62	2.03
Pae	1.053	1.225	2.30	2.60
Sen	0.445	0.431	1.63	1.99

2.3. Gamma-distributed time of egg-laying

Table B4. Maximum likelihood estimates of the shape and scale parameters of the Gamma-distributed timing of egg-laying for each experimental group, followed by the corresponding mean and standard deviation (SD) of the Gamma distribution.

food	size	shape	scale	mean	SD
Eco	1	4.55	0.212	2.52	0.453
Pae	1	5.37	0.211	2.66	0.488
Sen	1	4.53	0.175	2.32	0.372
Eco	10	5.73	0.171	2.53	0.409
Pae	10	4.17	0.253	2.58	0.516
Sen	10	5.95	0.136	2.34	0.332
Eco	25	3.93	0.274	2.63	0.543
Pae	25	5.54	0.208	2.68	0.491
Sen	25	5.03	0.164	2.36	0.368

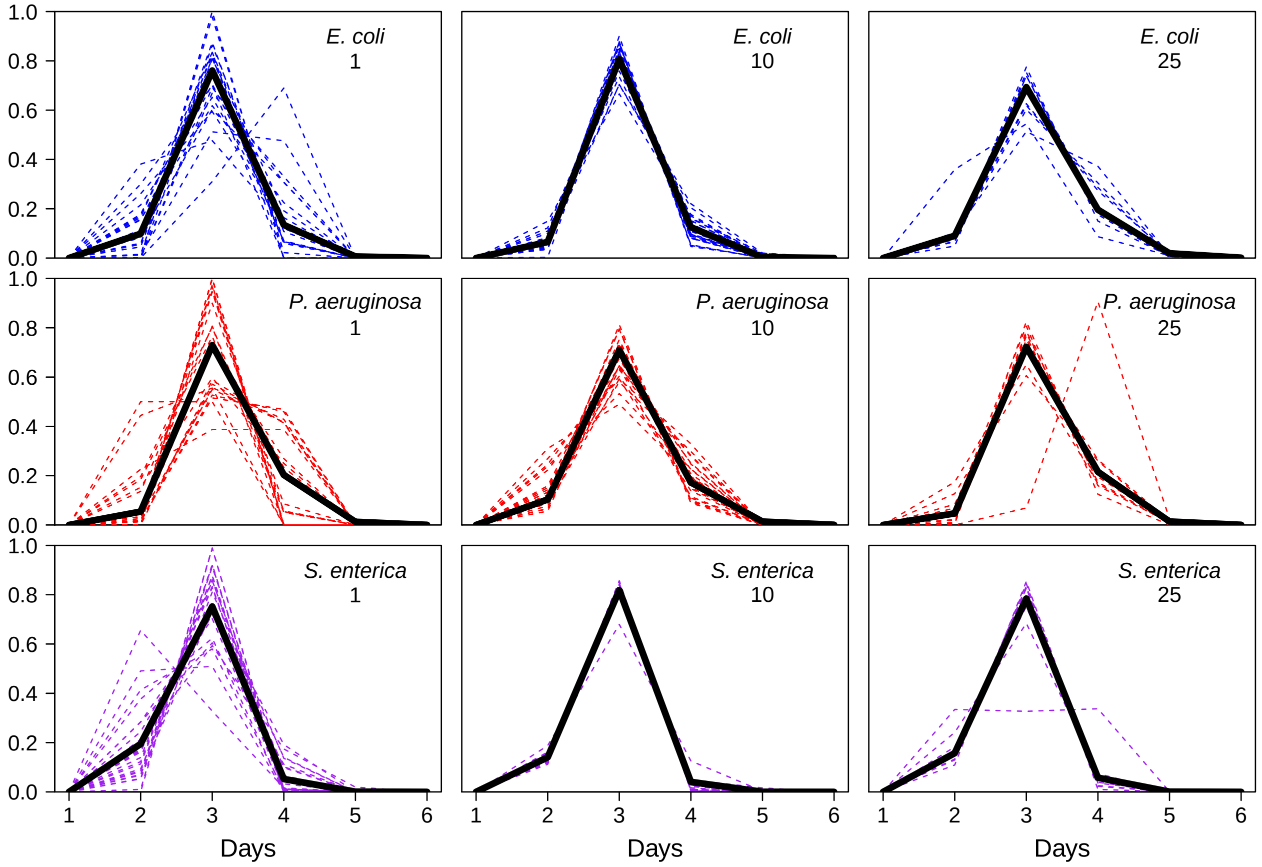

Figure B2. Comparison of the observed (dashed lines for each cohort) and predicted (solid lines) proportions of viable eggs laid each day.

2.4. Population dynamic model

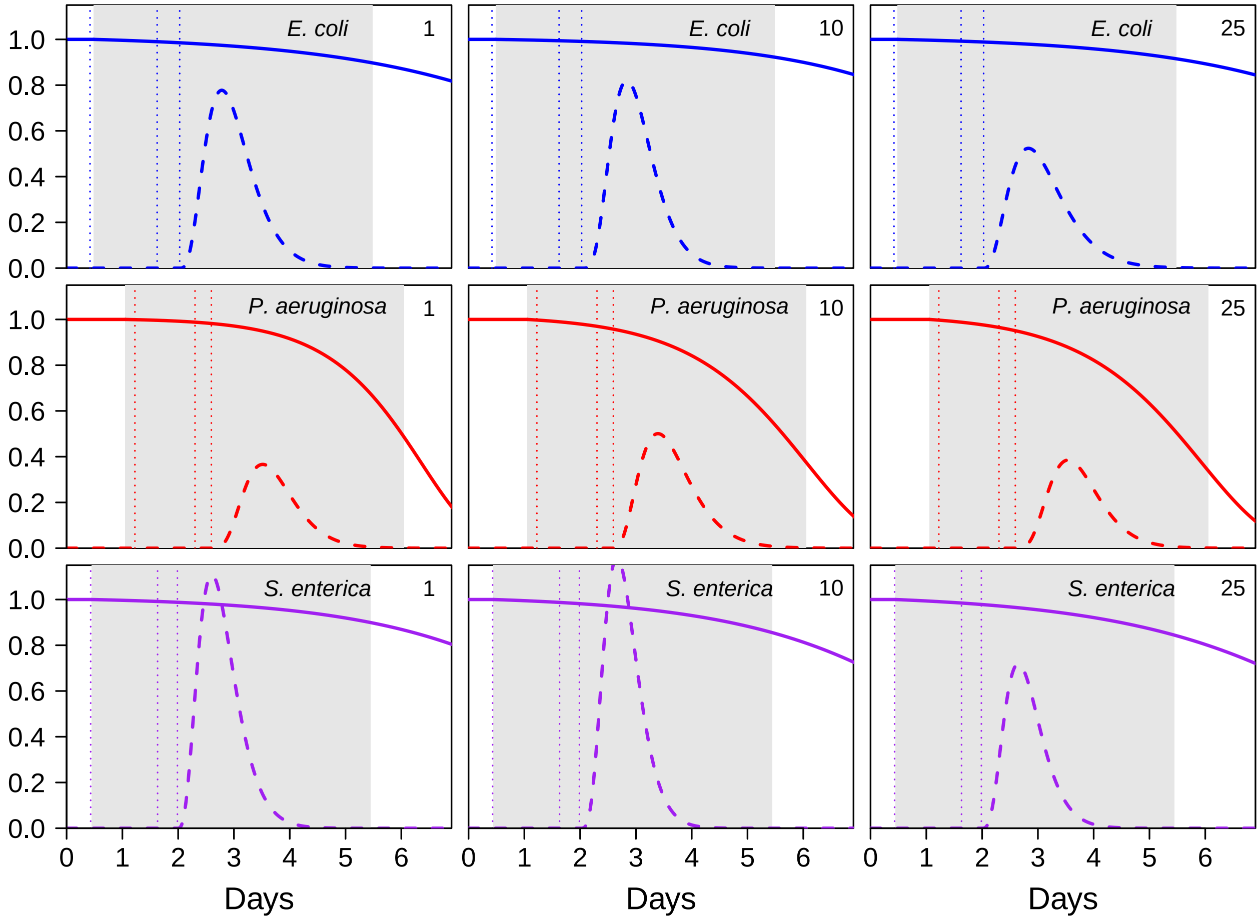

Figure B3. Combined graphs of the fitted survival and reproduction function. Solid line: survival curve, dashed line: egg laying, vertical dotted lines: morph transitions; shaded area: age span covered by the population growth experiment.

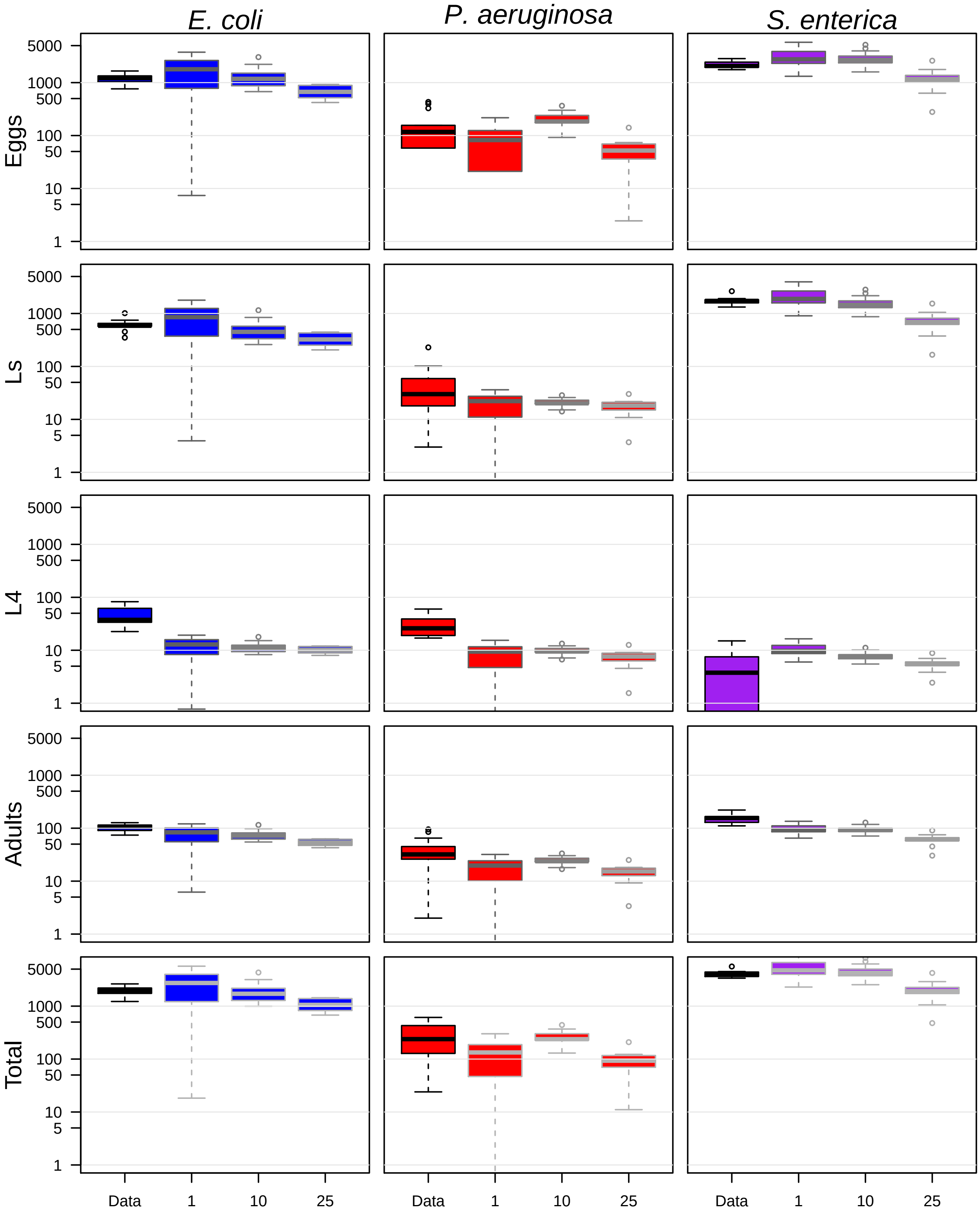

Figure B4. Effect of variability in fecundity on the predicted population composition after five days. In each panel, the first boxplot shows the data for each experimental group, and the other three boxplots show the distribution of predicted values across the empirical range of potential LRS for each cohort size (1, 10 and 25).

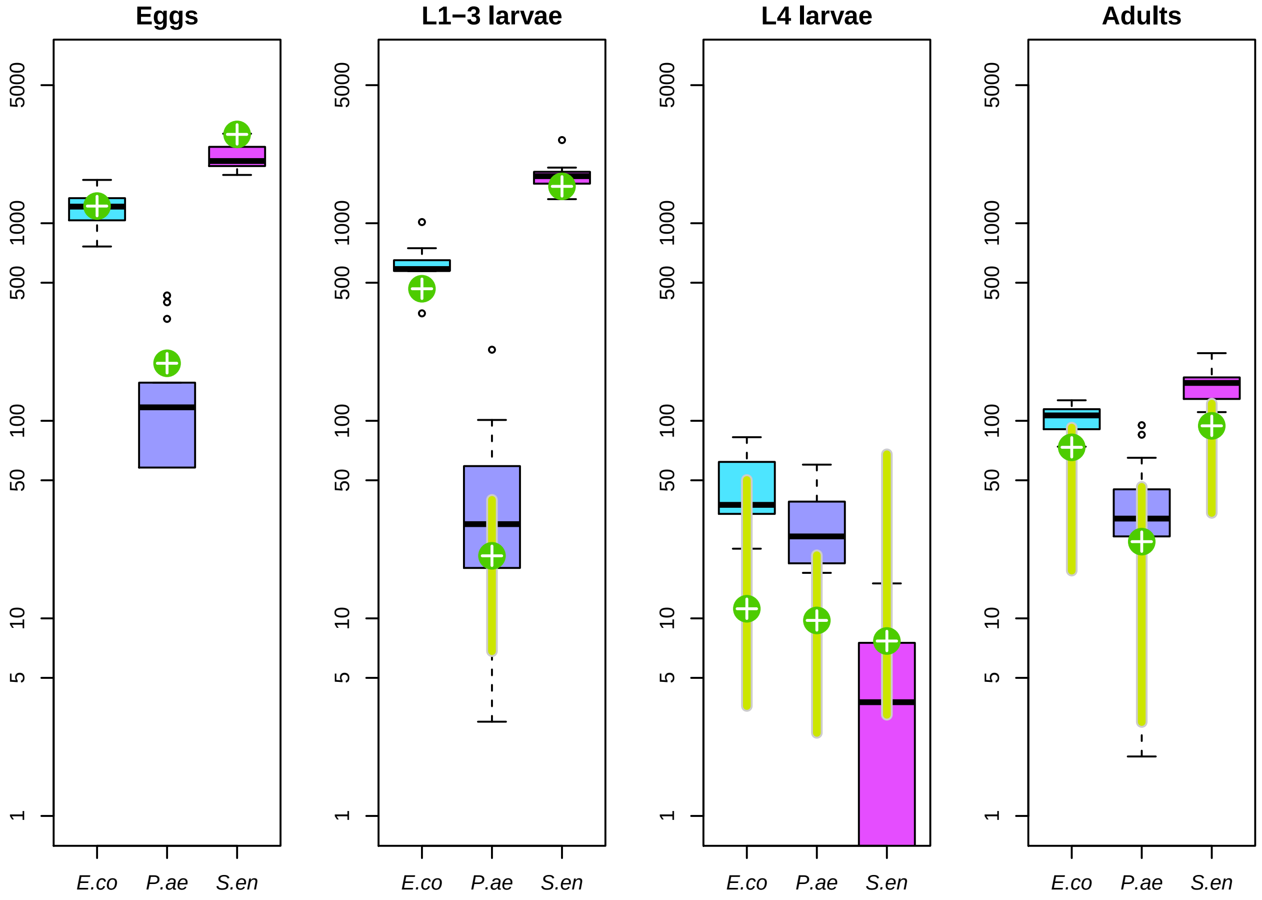

Figure B5. Sensitivity to development timing. As in Figure 4, the boxplots represent the data from the population growth experiment, and the green crosses show the predicted numbers of worms in each stage from the model parameterised with data from cohorts of 10 worms. In order to illustrate one of the factors that may have contributed to the relatively poor prediction of the numbers of L4 by the model (particularly in the *E. coli* and *P. aeruginosa* groups), we carried out a simple sensitivity analysis to the timing of the onset and duration of the L4 stage. In line with the range of developmental timings reported on Fig. 3, we varied the onset of L4 stage by +/- 12h around the mean value, and the average duration was multiplied by a factor ranging from 0.5 to 2. The green vertical bars show the range of the predicted numbers of worms in each stage when both parameters were varied simultaneously.

References

Arnold, Taylor A., and John W. Emerson. 2011. "Nonparametric Goodness-of-Fit Tests for Discrete Null Distributions." *The R Journal* 3 (2): 34–39. http://journal.r-project.org/archive/2011-2/RJournal_2011-2_Arnold+Emerson.pdf.
